# High-Resolution Analysis and Functional Mapping of Cleavage Sites and Substrate Proteins of Furin in the Human Proteome

**DOI:** 10.1371/journal.pone.0054290

**Published:** 2013-01-15

**Authors:** Sergey A. Shiryaev, Andrei V. Chernov, Vladislav S. Golubkov, Elliot R. Thomsen, Eugene Chudin, Mark S. Chee, Igor A. Kozlov, Alex Y. Strongin, Piotr Cieplak

**Affiliations:** 1 Infectious and Inflammatory Disease Center, Sanford-Burnham Medical Research Institute, La Jolla, California, United States of America; 2 Prognosys Biosciences Inc., La Jolla, California, United States of America; Stanford University, United States of America

## Abstract

**Background:**

There is a growing appreciation of the role of proteolytic processes in human health and disease, but tools for analysis of such processes on a proteome-wide scale are limited. Furin is a ubiquitous proprotein convertase that cleaves after basic residues and transforms secretory proproteins into biologically active proteins. Despite this important role, many furin substrates remain unknown in the human proteome.

**Methodology/Principal Findings:**

We devised an approach for proteinase target identification that combines an *in silico* discovery pipeline with highly multiplexed proteinase activity assays. We performed *in silico* analysis of the human proteome and identified over 1,050 secretory proteins as potential furin substrates. We then used a multiplexed protease assay to validate these tentative targets. The assay was carried out on over 3,260 overlapping peptides designed to represent P7-P1’ and P4-P4’ positions of furin cleavage sites in the candidate proteins. The obtained results greatly increased our knowledge of the unique cleavage preferences of furin, revealed the importance of both short-range (P4-P1) and long-range (P7-P6) interactions in defining furin cleavage specificity, demonstrated that the R-X-R/K/X-R↓ motif alone is insufficient for predicting furin proteolysis of the substrate, and identified ∼490 potential protein substrates of furin in the human proteome.

**Conclusions/Significance:**

The assignment of these substrates to cellular pathways suggests an important role of furin in development, including axonal guidance, cardiogenesis, and maintenance of stem cell pluripotency. The novel approach proposed in this study can be readily applied to other proteinases.

## Introduction

Many cellular proteins including growth factors, hormones, metalloproteinases and cell receptors are synthesized as inactive precursors. These precursors are transformed into functionally active proteins or peptides by the action of furin-like proteinases (proprotein convertases; PCs). The PC family comprises nine members: PC1/3, PC2, furin, PC4, PC5/6, Paired basic Amino acid Cleaving Enzyme 4 (PACE4), PC7, subtilisin/kexin isozyme-1 also known as Site-1 protease (SKI-1/S1p), and proprotein convertase subtilisin/kexin type 9 (PCSK9) [1], [2]. Because of overlapping substrate preferences and cell/tissue expression patterns, there is some redundancy in PC functionality. Nevertheless, individual PCs have certain distinct functions. For example, furin knockout is lethal in mice (3–4). Because of its ubiquity and biological importance, furin is the most studied enzyme of the PC family. A specialized serine endoproteinase, furin predominantly cleaves the multibasic consensus motifs R-X-R/K/X-R↓ [3]. Structurally and functionally, furin resembles its evolutionary precursor, the prohormone-processing kexin of the *Saccharomyces cerevisiae* yeast. Furin has been found to be expressed in all human tissues and cell lines examined to date, except colon carcinoma LoVo [4] and CHO-K1 RPE40 [5], [6] cells, and is mainly localized within the Golgi/trans-Golgi network secretory pathway.

In addition to normal cell functions, furin has also been implicated in many pathologies, including malignancy and infectious diseases, especially in the processing of bacterial and viral pathogens, such as anthrax and botulinum toxins, influenza A H5N1 (bird flu), flaviviruses, and Marburg and Ebola viruses [2], [3], [7], [8], [9], [10].

A more precise knowledge of the cleavage preferences of furin would assist in the identification of furin substrates in the human proteome and a better understanding of the functional significance of furin proteolysis in normal development relative to disease. A proteome-wide identification of furin substrates, however, requires both detailed knowledge of the cleavage preferences of this enzyme and a reliable *in silico* substrate selection pipeline. Therefore we believe that an efficient multi-step process for furin substrate identification should be based on a number of computational and experimental steps and should also include structural, spatial and temporal parameters rather than derived from the cleavage site sequence alone.

Current understanding of the cleavage preferences of furin is limited by a paucity of both known cleavage targets and data for synthetic peptide substrates. Currently, approximately 150 cleavage targets of furin are listed in several publicly available, albeit limited, databases including MEROPS [11], CutDB [12] and FurinDB [13]. However, these databases do not account for the existence of the multiple isoforms and multiple GI entries which, in fact, point to the same individual protein. The presence of both the multiple isoforms and GI entries makes the actual number of furin targets in these databases significantly higher. In addition, Turpeinen *et al*. [14] using genome-wide expression correlation data have recently identified 172 individual proteins, whose expression directly correlates with furin. However, only a limited number of these co-expressed proteins exhibit the multi-basic motif characteristics of the furin cleavage site. Current evidence suggests that furin exhibits narrow short-range cleavage specificity and a preference for Arg at P1, P2 and P4 residue positions [2], [3], [15]. The relative importance of other residue positions that are proximal to the substrate scissile bond is not well established. Normally, the proteins are assigned as furin targets by their sequence homology with the R-X-R/K/X-R↓ consensus cleavage sequence of furin.

The main purpose of our study was to develop the multi-step *in silico* pipeline that would be applicable for the identification of the protease substrates in the complex proteomes. As proof of principle, we developed this pipeline for furin and its substrates in the human proteome. To prove *in silico* findings we used a new highly multiplexed cleavage assay to clarify the cleavage preferences and provide detailed structural requirements for the interactions of furin with its protein cleavage targets. We combined *in silico* analysis with results from the multiplexed cleavage assay to reveal the importance of both short-range (P4-P1) and long-range (P7-P6) interactions in defining furin cleavage specificity. We demonstrated that because the P7, P6, P5, P3, and P1’-P4’ residue positions are also very important for furin, the presence of the R-X-R/K/X-R↓ multi-basic motif alone is an insufficient determinant for predicting furin proteolysis of the substrate protein. Based on the results of our study, we suggest that the human proteome includes approximately 490 potential furin cleavage proteins, which could be readily assigned to multiple cellular pathways. Our results also highlight the indispensable role furin plays in normal cell functions, especially in development. Additionally the *in silico* pipeline we developed can now be readily applied to other proteinases with known cleavage preferences.

## Results

### Initial Selection of Furin Cleavage Targets in the Human Proteome

**Figure 1:**
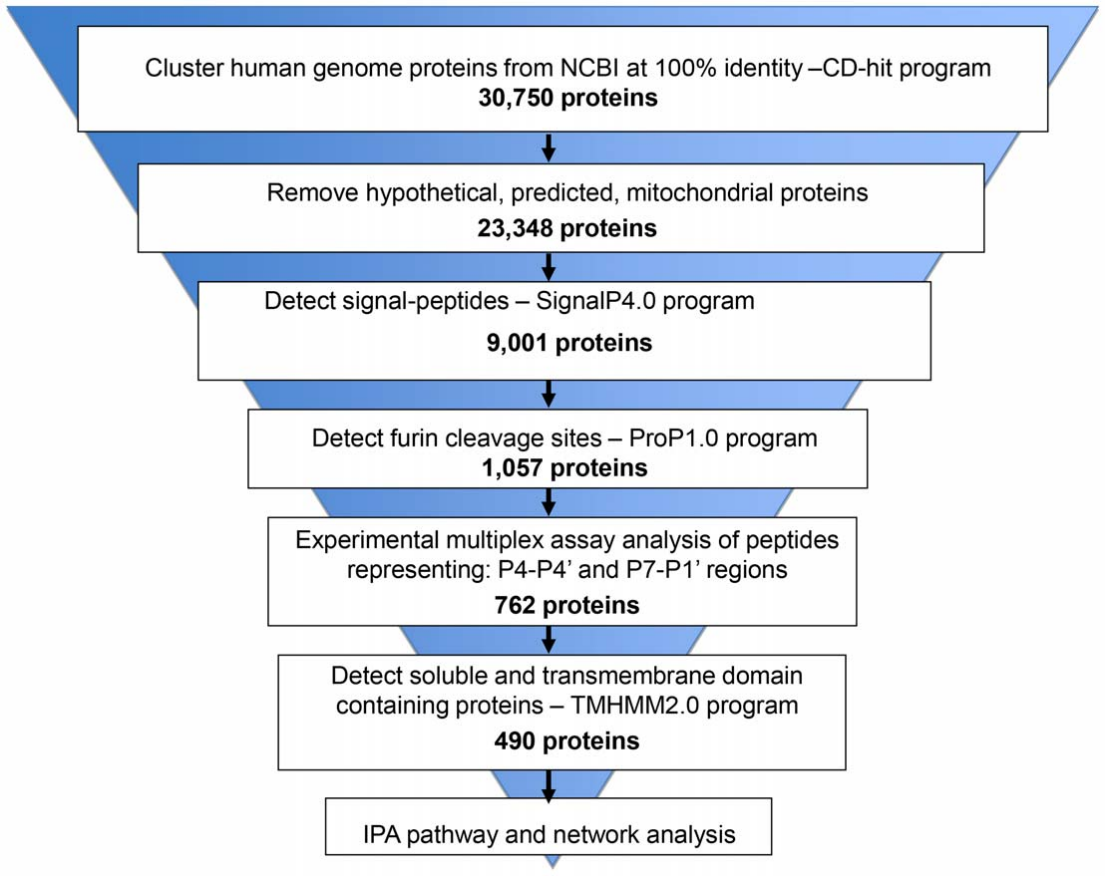
A flowchart representing the stepwise approach used to identify furin substrates in the human proteome.

We used a series of computational and experimental steps to identify physiologically-relevant furin substrates in the human proteome (Fig. 1). First, sequences of all human proteins were downloaded from National Center for Biotechnology Information (NCBI; ftp.ncbi.nih.gov/genomes/H_sapiens). To exclude redundant sequences, the downloaded sequences were clustered at a 100% identity level using CD-Hit [16]. This provided an initial set of 30,750 human proteins. Next, we removed protein sequences that were annotated as “hypothetical” and “predicted” by NCBI. We also removed mitochondrial proteins because they normally are not processed by furin [3]. The resulting set of 23,348 individual proteins was screened using ProP, an artificial neural network program [17], to identify sequences containing the cleavage motif (K/R)-(X)n-(K/R)↓ (where n is 0, 1, 2, 4 or 6 and X is any amino acid). The software identified 13,736 consensus furin cleavage motifs in 9,001 individual human proteins. We then made use of the fact that furin cleaves its protein targets within the secretory pathway. We identified the presence of the signal peptide sequence, an essential element of secretory proteins, in 1,057 out of 9,001 proteins, by using the SignalP4.0 neural network-based software [18]. These 1,057 secretory proteins exhibited over 1,400 potential furin cleavage sites or an average of 1.32 cleavage sites per protein. For comparison, there were 12,313 potential furin cleavage sites in 7,944 non-secretory proteins, or 1.55 sites per protein. This difference may suggest that secretory proteins have evolved to reduce the number of furin cleavage sites to escape from physiologically irrelevant furin proteolysis.

### Multiplexed Cleavage Assay

We measured experimentally the cleavage efficiency of the predicted furin cleavage sites using a multiplexed assay [19], [20] that utilizes peptide-cDNA fusions and high-throughput sequencing readout. This methodology greatly accelerates determination of the cleavage efficiency for the peptide substrates and provides an in-depth understanding of cleavage preferences.

We synthesized over 5,860 individual peptides, from which 3,260 peptides represented the P4-P4’ and P7-P1’ positions of the predicted and known furin cleavage sites. There was a three residue offset resulting in a five residue overlap between the P7-P1’ and P4-P4’ peptides. As a result, the effects of each of the P7-P4’ residue positions on the furin cleavage efficiency could be ascertained. The remaining 2,600 peptides were non-furin control sequences. These included peptide substrates of caspases, thrombin and enterokinase, and of proteinases from Dengue, West Nile, and hepatitis C viruses used in our earlier studies [20]. The resistance of these non-furin peptides to furin cleavage confirmed the selectivity and accuracy of our cleavage technology.

The pool of peptide-cDNA fusions was immobilized on magnetic beads using an affinity tag attached to the N-terminus of all peptides, and incubated in the presence of purified furin. Following proteolytic cleavage of the peptide portion of the fusion, the corresponding cDNA template was released in solution. The cleaved peptide substrates were identified by using next-generation sequencing for high-throughput digital analysis of the released cDNA [19]. Cleavage signals were expressed as Z-scores after comparing sequence counts in treated and untreated samples (see “Experimental Procedures”). The cleavage data obtained for 5,867 peptides are presented in Table S1.

### Analysis of Furin Cleavage Preferences

The peptides varied widely in magnitude of proteolysis: both the highly sensitive peptides (with Z-score >10) and peptides which resisted furin proteolysis (with Z-score below 3.5) were identified in the 5,867 peptide set. Table S2 contains information about the P7-P1’ and P4-P4’ sequences of the peptides for which experimental measurements were obtained, Z-scores as a function of time describing cleavage efficiency, the cleavage position, and the name of the protein from which the peptides were selected. This information is provided for each of 1,400 furin cleavage sites identified in 1,057 individual human proteins with the N-terminal predicted signal sequence.

**Figure 2:**
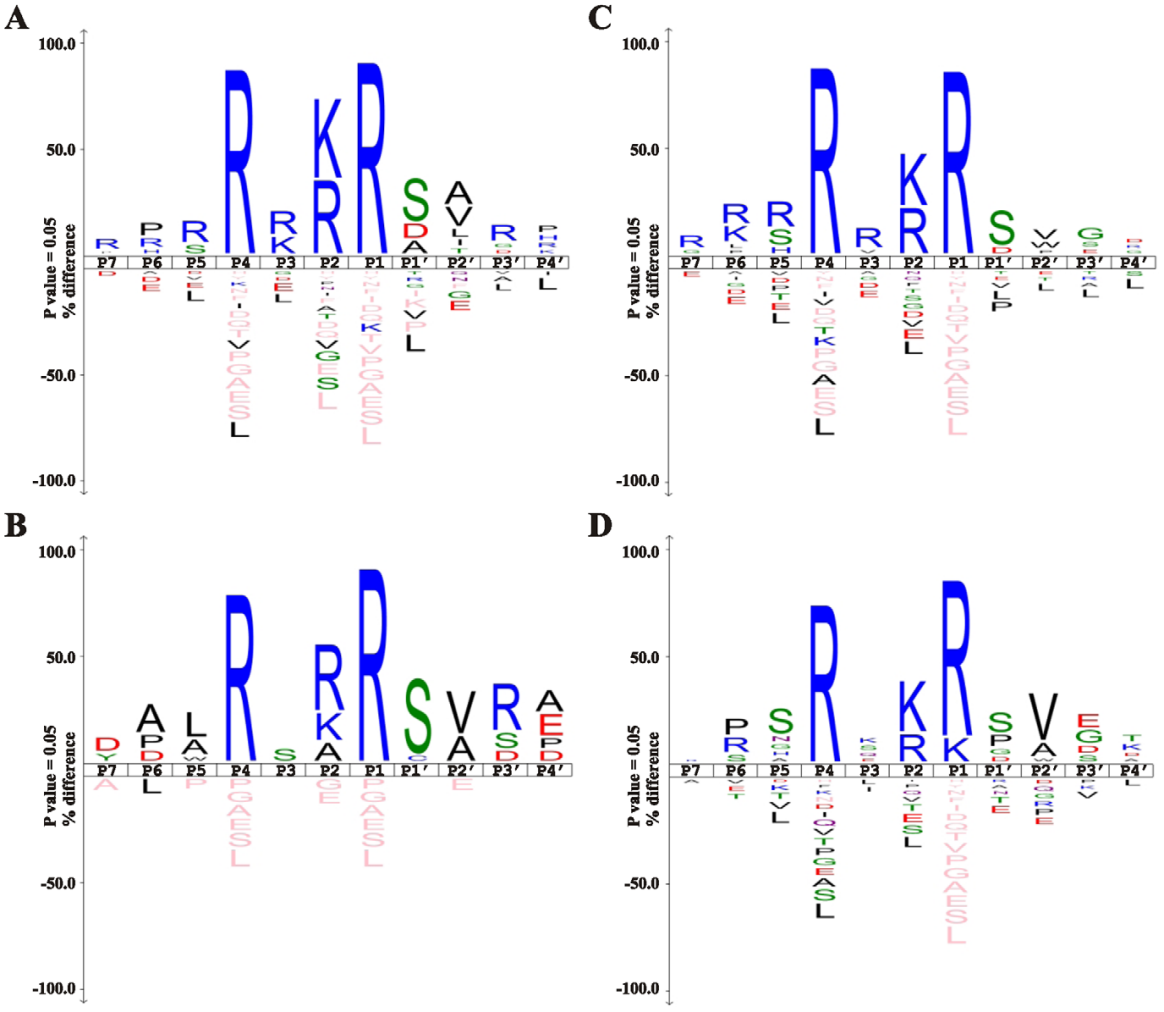
Frequency plot of the cleavage sequence of furin in an IceLogo format. The height of a character is proportional to the frequency of the amino acid residue at the individual position of the cleaved peptide and is normalized for the amino acid encoded in the entire human genome according to RefSeq. Because there was a five residue overlap between the P7-P1’ and P4-P4’ peptides from the individual cleavage site, the sequence logos represent the combined P7-P4’ peptide sequence. **A**. Peptide sequences with the Z-score over 3.5 for both P4-P4’ and P7-P1’ peptides (932 substrates). **B**. Peptide sequences with the Z-score for the P4-P4’ and P7-P1’ peptides over and below 3.5, respectively (64 substrates). **C**. Peptide sequences with the Z-score for the P4-P4’ and P7-P1’ peptides below and over 3.5, respectively (439 substrates). D. Peptide sequences for poor or no substrates with Z-score for the P4-P4’ and P7-P1’ peptides below 1.0 (391 sequences).

Based on these cleavage data, we established a direct relationship between the cleavage efficiency of furin and the amino acid sequence of the peptide substrates. This dependence is illustrated using sequence logos (Fig. 2) [21]. To perform an in-depth analysis of furin cleavage preferences, we divided the substrates into three groups. The first group comprised P7-P1’ and P4-P4’ peptides with Z-score >3.5 (932 substrates; Fig. 2A). The second group comprised P4-P4’ peptides with Z-score >3.5 and P7-P1’ peptides with Z-score <3.5 (64 substrates; Fig. 2B). This set revealed P7-P5 residues that decrease the efficiency of furin proteolysis. The third group comprised P4-P4’ peptides with the Z-score <3.5 and P7-P1’ peptides with Z-score >3.5 (439 substrates; Fig. 2C). This set revealed P2’-P4’ residues that decrease the efficiency of furin proteolysis. We would like to stress that the decrease of cleavage efficiency does not mean that a peptide is not cleaved, especially when the Z-scores is below 3.5 and above 1.0 values. Our results identify the (K/R)-(X)n-(K/R)↓ sequence, where n = 0, 1, 2, 4 or 6, and X is predominantly Arg or Lys, as a furin cleavage consensus motif (Fig. 2A). This motif is in agreement with the data that we and others reported earlier [1], [15]. In addition, our results revealed a high frequency of Asp at both P6 and P7 positions and of Leu, Ala and Trp at P5 in a subset of the least efficient substrates. The presence of P6 Ala or Pro also had a negative effect on cleavage efficiency (Fig. 2B). We recorded Ser as the most frequent residue at the P1’ substrate position, while predominantly Val and Ala were found at the P2’. However, Trp at the P2’ position was the the feature of the less efficient substrates (Fig. 2C). Our results also indicated that P3’ Asp, Arg and Ser, and P4’ Asp, Glu, Pro and Ala characterize good substrates while Gly and Glu at P3’ and Gly at P4’ were not well tolerated by furin. Fig. 2D demonstrates the peptides with the multi-basic motif with Z-score below 1.0 for both the P4-P4’ and P7-P1’ sequences. Apparently, this peptide set highlights that the presence of the multi-basic motif alone is an insufficient determinant for furin proteolysis. Thus, the presence of Pro at the P6, Asn, Gly and His at the P5, Gln and Glu at the P3, Pro and Gly at the P1’, Trp at the P2’, Glu and Gly at the P3’ and, finally, of Thr and Lys at the P4’ largely inactivated the cleavage of the multi-basic motif. Overall, our findings suggest an important role of long-range interactions and a significant effect of the residue positions which are distinct from the consensus multi-basic motif in defining the cleavage preferences of furin.

### Structural Requirements for Furin

**Figure 3:**
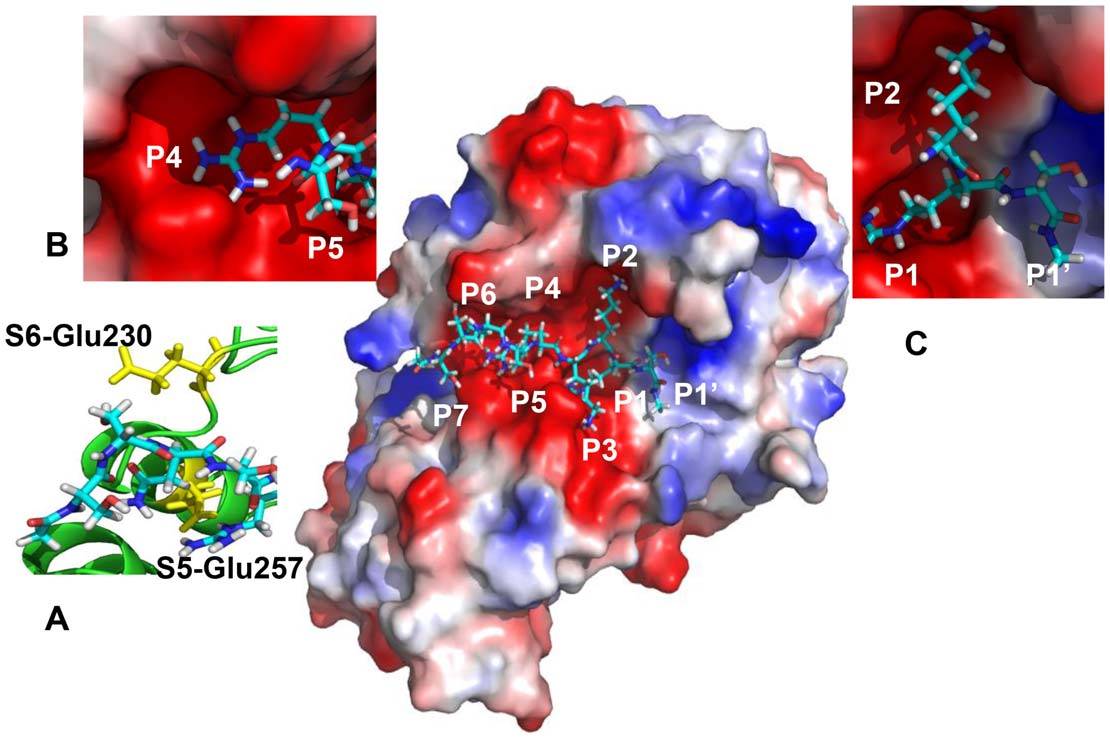
Structural modeling of furin with a protein substrate. **A**. The structure of furin with the SSNSRKRR↓S modeled substrate. The structure of furin-DEC complex (PDB 1P8J) was used as a template for modeling the protein substrate (shown as sticks) in the furin structure. The molecular surface of furin is colored according to the electrostatic potential (red, blue and white are negative, positive and neutral electrostatic potential values, respectively). Close-up on the left shows the S6 Glu230 and S5 Glu257 (yellow), which are critical for the interactions with the P6 and P5 substrate sub-sites. Close-ups (**B** and **C**) show in a more detail the P5-P4 and P2-P1’ residue positions in the furin active site.

We examined the structure of furin bound to the DEC inhibitor (PDB 1P8J) in order to elucidate structural elements that determine cleavage preferences, especially at the P7-P5 positions. The inhibitor coordinates served as a template for the binding of the peptide substrate with furin. The conformation of the peptide substrate SSNSRKRR↓S was modeled using the positions of the heavy atoms of the inhibitor and by incorporating the side-chains of amino acids residues into the structure. We optimized the final position of the substrate using molecular mechanical minimization and limited molecular dynamics simulations using AMBER11 software [22] (Fig. 3). The P1 and P1’ residues of the substrate were placed in those positions relative to the catalytic triad that are required for catalysis. The R↓S scissile bond was constrained during the molecular mechanical optimization procedure. Clearly, the negatively charged shallow groove of furin, including the S5-S7 sub-sites, can readily accept positively charged substrate Arg and Lys residues. Asp at both P7 and P6 of the substrate is likely to interfere with the negatively charged Glu230 and Glu257 of furin (Fig. 3), thus affecting the productive conformation and reducing the cleavage efficiency.

### Proteome-wide Prediction of Furin Substrates

In order to identify the most probable protein substrates of furin, we restricted our analysis to the most efficiently cleaved peptide sequences (Z-score >3.5 in the 7.5 min reactions for both P7-P1’ and P4-P4’ substrates). Applying this stringent criterion, we selected 762 unique human GI protein sequence entries, containing 932 predicted cleavage sites, from our initial set of 1,057 GI entries.

Next, we selected membrane proteins with the furin cleavage motif localized in the ectodomain moiety, which is accessible to furin proteolysis, and filtered out those membrane proteins in which the putative cleavage sites are in the intramembrane segment or the cytoplasmic tail domain, both of which are inaccessible to furin. The presence and the position of the transmembrane domain region were predicted in the 762 proteins using the TMHMM2.0 program [23]. Our final set of potential furin proteolysis targets comprised 240 unique GI membrane protein sequences with the furin cleavage motif in the ectodomain and 358 unique GI soluble secretory protein sequences (Table 1). These 598 unique human GI protein sequence entries contained a total of 751 furin cleavage sites. This dataset, however, included multiple isoforms and “long” and “short” splice variants of the individual proteins. Removing these isoforms and splice variants resulted in the 490 unique proteins (184 membrane and 306 soluble proteins, respectively), which are the most probable furin cleavage targets in the human proteome (Table S3). Classification of the predicted furin protein substrates is included Table 2.

**Table 1:** Counts of probable protein substrates of furin and cleavage sites determined by our computational-experimental approach.

Membrane- associated protein	Location of the furin cleavage position	Number of unique GI protein sequences	Number of cleavage sites
Yes	Cytoplasm/intramembrane	164	181
Yes	Extracellular (ectodomain)	240	298
No (soluble proteins)	-	358	453

There are over 490 probable secretory protein substrates (or 598 GI protein sequence entries) in total (soluble proteins and membrane proteins with detected cleavage positions located in the extracellular domain). There are a total of 751 detected cleavage sites in the GI protein sequence entries which correspond to these 490 proteins.

**Table 2:** Classification of the predicted furin protein substrates (based on the 598 GI protein sequences).

Protein type	Total number
Proteinases and inhibitors	97
Extracellular matrix proteins	128
Receptors	137
Growth factors and hormones	80
Serum proteins	24
Enzymes	42
Others	90

It should be noted that our pipeline predicted several proteins that clearly do not belong to furin substrates. Because of the partially overlapping cleavage preferences of trypsin-like proteinases and furin, these proteins [complement component 2 isoform 1 preproprotein (gi: 14550407), complement component 3B preproprotein (gi: 178557739), complement component A4 preproprotein (gi:67190748), coagulation factor V precursor (gi:105990535), complement component 4B preproprotein (gi:178557739), coagulation factor VIII isoform a precursor (gi:4503647), complement component 8, alpha polypeptide precursor (gi:4557389), coagulation factor II preproprotein (gi:4503635), complement factor properdin precursor (gi:223671861), and complement component 8, beta polypeptide precursor (gi:4557391)] exhibit the furin cleavage motif. However, these coagulation and complement system proteins normally circulate as inactive precursors in the blood and then are activated by specialized proteinases in the system, rather than by furin, to initiate an amplifying cascade of further cleavages [24], [25], [26].

### Comparison with Other Databases

There were some disagreements between the existing databases and our results. For example, our selection process suggests that three proteins listed as furin targets by MEROPS lack a signal peptide [TNFS12-TNSF13 protein (gi: 26051250), TNSF13B protein (gi: 5730097) and leukocyte cell-derived chemotaxin 1 isoform 1 precursor (gi: 5901932)] and, as result, they cannot be cleaved by furin under the physiologically relevant conditions. In an additional 15 proteins, the presence of the furin cleavage motif was not recognized by the ProP program. Therefore, we did not synthesize and test the peptides that corresponded to the folowing proteins: integrin αV isoform 1 precursor (gi: 4504763), type IV collagenase isoform a preproprotein (gi: 11342666), proprotein convertase subtilisin/kexin type 9 preproprotein (gi: 31317307), lactase-phlorizin hydrolase preproprotein (gi: 32481206), natriuretic peptides B preproprotein (gi: 4505433), vascular endothelial growth factor C preproprotein (gi: 4885653), beta-secretase 1 isoform A preproprotein (gi: 6912266), integrin alpha-5 precursor (gi: 56237029), proline-rich protein BstNI subfamily 3 precursor (gi: 117306167), platelet derived growth factor D isoform 1 precursor (gi: 13376808), gastrin preproprotein (gi: 4503923), pancreatic polypeptide preproprotein (gi: 4506033), proenkephalin (gi: 5453876), pro-opio melanocortin preproprotein (gi: 80861463), platelet-derived growth factor C precursor (gi: 9994187) and stromelysin-1 preproprotein (gi: 4505217). Two additional secretory proteins from MEROPS [proheparin-binding EGF-like growth factor precursor (gi: 4503413) and vascular endothelial growth factor D preproprotein (gi: 4758378)] were not included in our set of the most probable cleavage targets of furin because both the corresponding P7-P1’ and P4-P4’ peptides with Pro at the P3 were cleaved inefficiently in our *in vitro* cleavage tests (Z-score <3.5). Similarly, 11 proteins that are listed as furin targets in the CutDB database were not included in our final protein set. For example, according to ProP, there was no identifiable furin cleavage motif in urotensin-2 isoform b preproprotein (gi: 5803209) and there was no recognizable signal peptide sequence in zona pellucida sperm-binding protein 3 isoform 2 (gi: 38327649). An additional 9 proteins were in common with those found in the MEROPS database and were already eliminated (gi: 5901932, 11342666, 31317307, 32481206, 4505433, 4885653, 6912266, 56237029, and 4505217). We also confirmed that the furin targets listed in the FurinDB database [13] are also present in our dataset. We also determined that not all of the proteins co-expressing with furin [14] exhibit the furin cleavage motif and, as a result, these proteins cannot be the targets of furin proteolysis. Although we cannot be certain that all the excluded proteins are not targets of furin, we chose to err on the side of stringency to minimize the number of potential false positives.

Nevertheless, the pipeline we designed identified a majority of proteins which are widely recognized as furin targets such as members of the matrix metalloproteinase (MMP), ADAM (A Disintegrin And Metalloprotease) and ADAMTS (A Disintegrin and Metalloproteinase with a Thrombospondin repeat) proteinase families, integrins, cadherins and other cell adhesion and signaling receptors, Notch, interleukins and growth factors, hormones, selectins and semaphorins, extracellular matrix proteins including collagens, tenascins, laminins, aggrecan, brevican and dystroglycan, bone morphogenic proteins, proteinases and their protein inhibitors and multiple additional furin substrates. However, to the best of our knowledge, the functionality of multiple additional proteins, including hedgehog-interacting protein, peroxidasin, endothelial lipase and tsukushin, was never linked to their respective precursors by furin.

### Exemplary Substrate Validation

**Figure 4:**
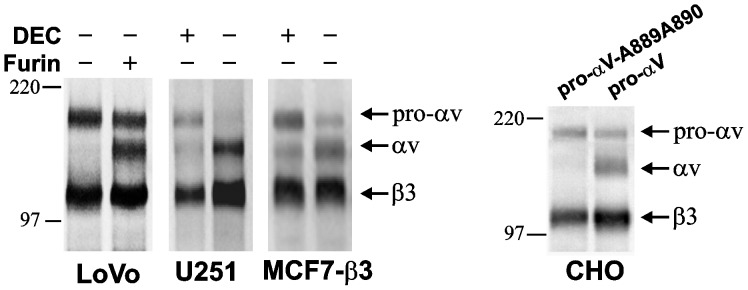
Furin processing of integrin αV. The original glioma U251 cells, breast carcinoma MCF-7 cells with the enforced expression of the β3 integrin subunit (MCF7-β3), colon carcinoma LoVo cells with and without the enforced expression of furin and CHO cells with the enforced expression of the integrin αV precursor (pro-αV) or the furin-resistant pro-αV mutant (pro-αV-A889A890) were cell-surface biotinylated and then lysed. The lysates were precipitated using the αVβ3 antibody LM609 and analyzed by Western blotting with Extravidin-horseradish peroxidase. Where indicated, cells were co-incubated for 16–18 h prior to biotinylation with a synthetic furin inhibitor (DEC; 50 μM).

As a result of our studies it now becomes evident that the presence of the basic motif is insufficient for describing furin cleavage requirements. To test the validity of our predictions, we selected integrin αV as a furin substrate. According to our peptide cleavage data, furin should cleave the Arg890-Asp891 scissile bond in the single-chain integrin αV precursor. The P7-P4’ amino acids sequence in the vicinity of the cleavage site is as follows: DHLITKR↓DLAL. This cleavage should generate the heavy and light α-chains connected by a disulfide bridge between Cys852 and Cys904. In our studies we used glioma U251, breast carcinoma MCF-7 and colon carcinoma LoVo cells. Because of two frame-shift mutations in the furin gene, LoVo cells do not exhibit catalytically active furin in contrast with U251 and MCF-7 cells. [4] LoVo and U251 cells, however, synthesize significant amounts of the individual αV and β3 integrin subunits, the interaction of which results in the αVβ3 integrin heterodimer [27], [28]. In turn, the level of the β3 integrin subunit is low in MCF-7 cells [29]. Because of these limitations, we used MCF7 cells with the enforced expression of β3 integrin, LoVo cells with the enforced expression of furin and the original U251 cells in our study. In addition, we also used CHO cells transfected either with the wild-type integrin αV or the furin-resistant mutant integrin αV constructs. In the latter, Ala-889 and Ala-890 substituted for Lys-889 and Arg-890 of the furin cleavage motif, thus rendering the mutant resistant to furin proteolysis. Where indicated in Fig. 4, a potent inhibitor of furin (DEC), was added to the cells to repress the furin activity and, consequently, the processing of the single-chain integrin αV precursor by furin. To facilitate the analysis of cell surface-associated integrin αVβ3, cells were surface-biotinylated using membrane-impermeable biotin. The cells were then lysed and cell-surface integrin αVβ3 was pulled-down using the αVβ3 LM609 antibody. Precipitated samples were analyzed by Western blotting. The biotin-labeled αV and β3 integrin subunits were visualized using Extravidin conjugated with horseradish peroxidase and a peroxidase substrate (TMB/M).

In agreement with our expectations, in U251 and MCF7-β3 cells a synthetic inhibitor of furin (DEC) significantly repressed the furin-dependent conversion of the single-chain integrin αV precursor into the mature αV integrin. Consistently, the αV integrin precursor alone was observed in furin-deficient LoVo cells. In turn, the αV integrin precursor was readily converted into mature αV integrin in LoVo cells with the enforced expression of active furin. In accord with these results, the αV integrin precursor was partially transformed into the mature αV integrin in CHO cells. In contrast, the inactivation of the furin cleavage site by alanine mutagenesis made the resulting Ala889-Ala890 αV integrin mutant fully resistant to furin processing in CHO cells. These mutagenesis results have never been included in our earlier reports.

### Role of the Secondary Structure of the Cleavage Region in Furin Proteolysis

In the Protein Data Bank (PDB) [30], there are crystal structures of 254 proteins from the 490 furin protein cleavage targets we predicted. However, except a few structures, all of others have been determined using the mature proteins which have been processed by furin rather than the precursor proteins with the furin cleavage sites [31]. The analysis of these few remaining structures revealed that the cleavage site, as expected, are located in the unstructured loops which are presented on the protein surface. These include, for example: Notch1 preproprotein (PDB id: 3V79), complement component C2A (2I6S), cathepsin C isoform a preproprotein (3PDF), netrin G2 (3ZYI), UL16 binding protein (1KCG), fibroblast growth factor 23 precursor ((2P39), hemopexin precursor (1QJS), plasma carboxypeptidase B2 isoform b (3D68), peptide deformylase (3G5P) and others. In many other cases, for example in the structure of αV integrin protein discussed above, (PDB id: 3IJE), an entire loop Q869-G897 with the KR890-D cleavage site is missing in the solved crystal structure.

### Proteolytic Network

**Figure 5:**
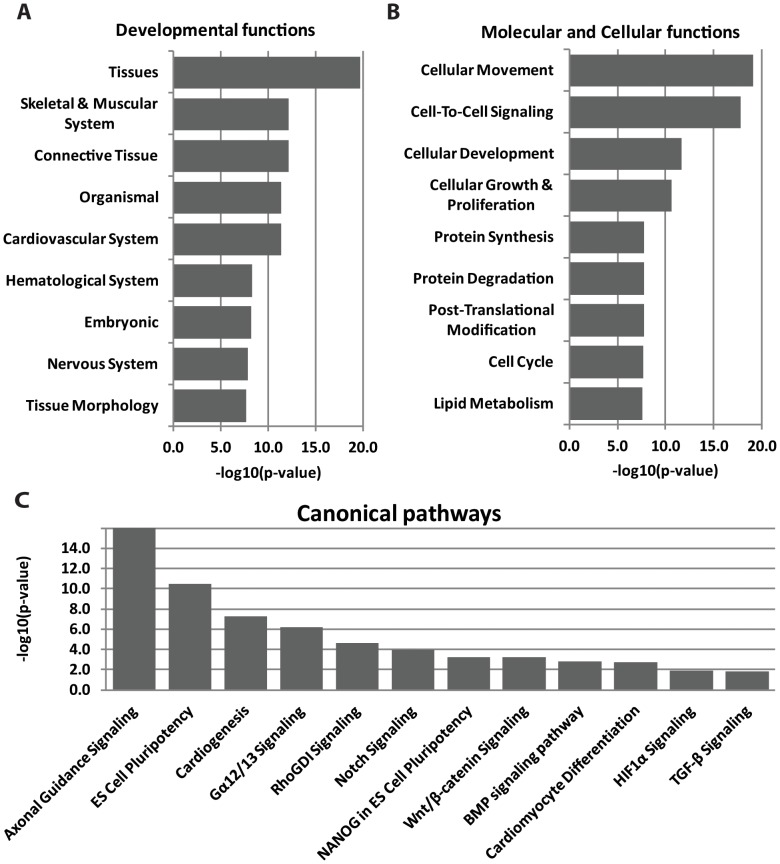
Functional analysis of predicted furin human substrates. Ingenuity Pathway Analysis was used to identify the most significant developmental functions (**A**), molecular and cellular functions (**B**) and canonical pathways (**C**) associated with the predicted furin protein substrates. Right-tailed Fisher exact test was used to calculate a p-value. Only pathways with P<0.05 are shown.

**Figure 6:**
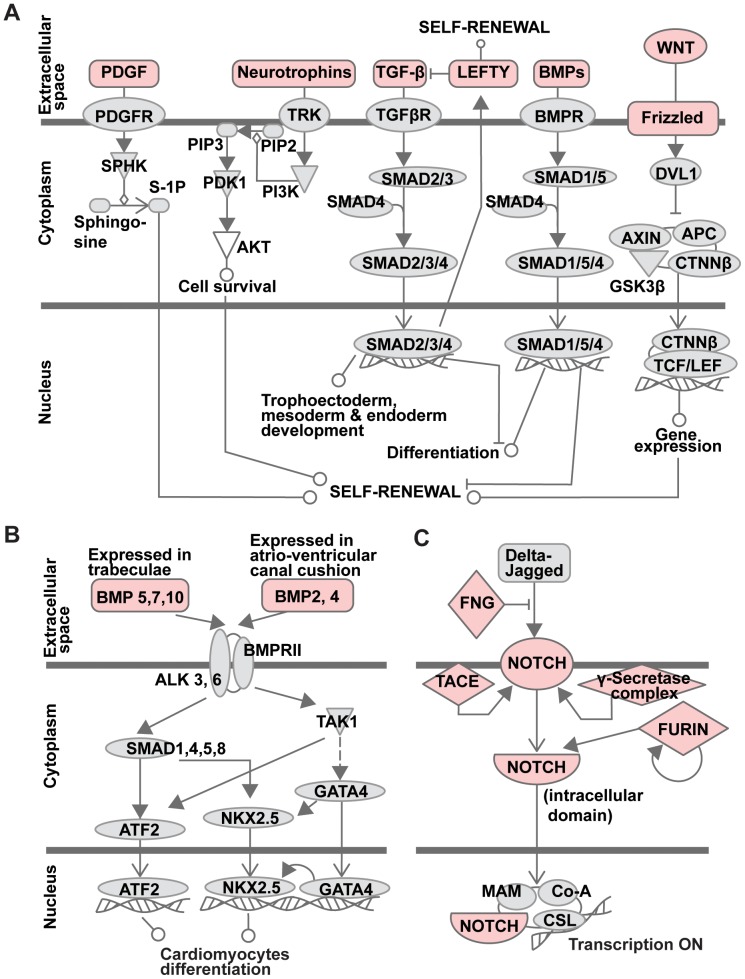
Examples of canonical pathways which are likely to be regulated by furin proteolysis. **A**. Pluripotency in human embryonic stem cells; **B**. the bone morphogenic protein (BMP) pathway in cardiomyocyte differentiation; **C**. Notch signaling pathway. Furin substrates are pink.

The existence of a significant number of predicted substrate proteins of furin proteolysis in the human proteome allowed us to assess which signaling and metabolic pathways, molecular networks, and biological processes might be influenced by furin activity. We found, using Ingenuity Pathway Analysis [32] (http://www.ingenuity.com), that at least 478 of our predicted 598 individual GI protein sequences are members of known canonical pathways. Next, a systems biology meta-analysis was conducted using Ingenuity Pathway Analysis to assess the significance of furin proteolysis in pathways regulation. The significance of the association between the proteins in our data set and the canonical pathway was measured based on p-values calculated using right-tailed Fisher’s exact test. Only pathways with p<0.05 were considered for further analysis. We determined that a broad range of developmental functions, including development of tissues, musculoskeletal and nervous systems, as well as embryogenesis are potentially dependent on furin proteolysis (Fig. 5A). Cellular movement, cell-to-cell signaling and metabolism of lipids are among the top molecular and cellular functions, which are likely to be regulated by furin (Fig. 5B). It also appears that furin plays an important role in protein degradation *via* activation of the ADAM/ADAMTS families of proteinases and affects proteins involved in drug metabolism. Canonical pathways related to neurogenesis, cardiogenesis and maintenance of stem cell pluripotency have the largest number of components that are predicted to be furin cleavage targets (Fig. 5C). Notably, human embryonic stem cells pluripotency pathway involves at least 22 furin protein substrates, including members of Smad, Wnt and lipid signaling pathways (Fig. 6A). Multiple bone morphogenesis proteins (BMPs) including BMP1, 2, 3, 4, 7, 8A, 8B and 10, which are in our 598 individual GI protein sequences set and which are important for heart, neural system, cartilage and post-natal bone development, are also potential targets of furin cleavage (Fig. 6B). Our predictions indicate that NOTCH1, NOTCH2 and NOTCH4, the signaling of which is crucial for cardiogenesis, neurogenesis and stem cell differentiation, are targets of furin, which agrees with previous experimental observations [33]. Overall, our analysis predicts that furin proteolysis plays an important role in multiple biological processes and especially in the development of nervous, connective and cardiovascular systems in embryogenesis.

## Discussion

Furin, a member of the proprotein convertase family, is a multi-domain proteinase, the catalytic domain of which is similar in structure to bacterial subtilisin. Furin functions in the Golgi apparatus, in the secretory vesicles and, potentially, also on cell surfaces. This unique specificity proteinase cleaves after basic residues many functionally important cellular proteins, including soluble and membrane-tethered metalloproteinases, integrins, signaling receptors, growth factors, hormones and neuropeptides, and transforms them into their respective mature forms [34]. In addition to processing cellular precursor proteins, furin is also exploited by numerous bacterial and viral pathogens. Pathogenic viruses and bacterial toxins employ host furin to become fully functional and to allow entry into host cells and to cause disease onset.

In this study, we developed an approach for proteinase target identification and used it to identify approximately 490 probable targets of furin proteolysis in the human proteome (Table 1). This number significantly exceeds the number of previously identified and experimentally confirmed substrates and demonstrates the power of our highly multiplexed, proteome-wide approach. We confirmed that a majority of the known furin substrates reported in CutDB and Merops databases were identified by our pipeline and included in our furin substrate dataset.

Furthermore, since we have accomplished our experimental studies and our manuscript was under preparation, others obtained valuable experimental confirmations of our predictions. For example, it has been recently shown in the direct laboratory experiments that glypican [35], bone morphogenetic protein 9 and 4 [36], growth differentiation factor 11 [37], endothelin and adrenomedullin [38], Kazal type 5 [39], and angiopoietin-like protein 4 [40] are furin substrates as we predicted by using our in silico pipeline. We are confident that additional experimental confirmations will follow in a near future. In principle, our approach to discover proteinase targets can be applied to other proteinases in eukaryotic, prokaryotic and viral proteomes, and may ultimately enable the identification of new therapeutic targets.

We also established a method for measuring the efficiency of furin proteolysis, identified the effect of the residues at P7-P4’ positions on the overall cleavage efficiency of the substrate, and determined the precise cleavage preferences of furin. We demonstrated that the multi-basic amino acid motif is not a sufficient requirement for the efficient furin proteolysis of the substrate protein. We showed that amino acids located at the P7, P6, P5, P3, and P1’-P4’ oppositions are very important for modulating furin cleavage efficiency. This preference information can be used in the design of inhibitors, which, especially for proteinase antagonists, frequently starts with a known substrate. For example, because several furin-like individual PCs and furin exhibit similar P4-P1 preferences, furin inhibitors with P4-P1 occupancy alone will cross-react with PCs, which is a known phenomenon [41]. However, our results indicate that cross-reactivity with PCs might be decreased by designing inhibitors that occupy not only the S4-S1 but also the S6-S7 sub-sites in the furin active site. This specificity requirement, however, may increase the size of furin inhibitors and, as a result, negatively affect their drug-likeness parameters including cell permeability. Nevertheless, inhibitors designed in this way could be useful research tools.

Because of its high positive charge, the multi-basic cleavage motif is predominantly localized on the protein surface and is readily accessible. We initiated the analysis of the structural parameters determining accessibility of the cleavage site to a proteinase. The structural parameters may play a significant role in affecting the proteolysis efficiency [42]. Accordingly, our pipeline could be improved by including a structural analysis of the cleavage site vicinity. This task could be accomplished by either including the prediction of the secondary structure elements involving the cleavage sites [43], by inspecting available experimental structures from the Protein Data Bank [30], or by analyzing three-dimensional protein models built using homology modeling approaches [44]. The results obtained in this study can be used for both development of a better mathematical model for detecting proteinase cleavage sites and for improvement of the existing methods of cleavage site prediction [17], [18].

Our combined *in vitro* and *in silico* analysis culminated with the assignment of probable furin substrates to key signaling and metabolic pathways, molecular networks and biological processes, suggesting directly that furin proteolysis plays a highly significant role in normal development and embryogenesis, and, specifically, in regulating axonal guidance and cardiogenesis, and maintenance of stem cell pluripotency. These findings are consistent with the phenotype observed in the furin knockout mice [45]. We believe that our results can help guide experiments to elucidate the molecular mechanisms of furin-associated human pathologies, which may lead to the design of new pharmacological interventions.

## Materials and Methods

### Reagents

Reagents were purchased from Sigma-Aldrich unless indicated otherwise. Furin and the two-component NS2B-NS3 proteinase from Dengue and West Nile viruses were isolated and characterized as described earlier [46], [47], [48].

### Peptide Synthesis and Cleavage Assay

Peptide synthesis and both the precise methodology of the *in vitro* cleavage assay and of measuring and registering the peptide cleavage level of cDNA-peptide fusions were described earlier [19]. In our current study, we produced 5,867 peptide set that contained 8-mer peptide sequences. The first group consisted of 3,260 overlapping peptide sequences that represented the P4-P4’ and P7-P1’ positions of the potential furin cleavage sites in human proteins. The second group consisted of over 2,600 control peptides. This group included the control peptides, which were substrates of caspases (CS – see Table S1), Dengue virus derived peptides (DV), substrates of enterokinase (EK), a control set of known furin substrates (FU), thrombin substrates (TR), TEV proteinase substrates (TV), substrates of NS2B-NS3 and NS3/4A proteinases from West Nile (WN) and hepatitis C viruses (HC), and simple, low complexity peptides serving as a negative set (NG). The synthesized peptides contained constant N-terminal and C-terminal sequences (Cys-Ala and Ala-Gly-Asn-Ala-Ser-Ala-Ser-Ala, respectively) flanking an 8-residue variable, furin-specific sequence. These peptides are conjugated to a cDNA oligonucleotide.

### Proteinase Assay

The cDNA-peptides fusions were immobilized on magnetic beads *via* the N-terminus and treated with furin for 7.5, 15, or 30 min at a 1∶10 enzyme-substrate ratio in 0.01 ml reactions containing 50 mM HEPES, pH 7.5, 1 mM CaCl_2_ and 100 mM NaCl. Reactions without furin were used as a negative control. DNA molecules released by peptide cleavage were collected from each sample and sequenced following the attachment of adapter sequences by PCR [49].

### Cleavage Data Analysis

Peptide abundance in solution was quantified by counts of DNA reads corresponding to each peptide sequence. The cleavage levels were estimated by comparing the log-transformed counts in the proteinase-treated *versus* the untreated samples. We used a locally weighted scatter plot smoothing fit as implemented in the lowess (locally weighted scatterplot smoothing) function from the statistical analysis package R to adjust for sequence-specific variance in abundance levels. The residuals of the fit were modeled as arising from a mixture of two distributions with different means. The main peak with mean of residuals equal to 0 (due to lowess robustness) corresponded to the intact peptides and the second peak with positive mean corresponded to cleaved peptides. The robust standard deviation of residuals was computed using the median absolute deviation estimator after which residuals were converted to Z-scores. After this transformation, Z-scores of intact peptides were assumed to have a standard normal distribution. Statistical significance was inferred by converting Z-scores to p-values and adjusting for multiple hypothesis testing using false discovery rate [50]. We chose to reject the hypothesis of no cleavage at Z-score >3.5, which corresponds to nominal p<0.0002 and false discovery rate <0.01. When in question, the identity of the scissile bonds in certain peptides was confirmed in the cleavage experiments followed by mass-spectrometry analysis of the digest.

The sequence logos were obtained by calculating cleavage efficiency over the entire set of substrates and then selecting the substrates with efficiency equal or above the Z-score = 3.5 threshold. These substrates were considered susceptible to furin proteolysis. Substrates with cleavage efficiency below the threshold were considered resistant to furin proteolysis. The resulting logos were created by the web-based IceLogo program [21].

### Cells, Integrin Pull-down and Western Blotting

Original human glioma U251, breast carcinoma MCF-7 and colon carcinoma LoVo and Chinese hamster ovary CHO cells were obtained from ATCC. MCF-7 cells transfected with the full-length human β3 integrin subunit, LoVo cells transfected with human furin and CHO cells transfected with the full-length human αV integrin subunit and with the mutant αV integrin subunit with the inactivated furin cleavage site were prepared and characterized earlier [29], [46], [51], [52], [53]. In the furin-resistant αV integrin mutant, Ala-889 and Ala-890 substituted for Lys-889 and Arg-890 of the furin cleavage motif. Where indicated in our figures and figure legends, cells were incubated for 16–18 h with a furin inhibitor decanoyl-Arg-Val-Lys-Arg-chloromethylketone (50 μM; DEC; Bachem). For the analysis of the cell surface-associated integrin αVβ3, cells were surface biotinylated using membrane-impermeable EZ-Link sulfo-NHS-LC-biotin (sulfosuccinimidyl-6-(biotinamido)hexanoate; 0.1 mg/ml; Pierce). Cells were lysed in 20 mM Tris-HCl, 150 mM NaCl, 1% deoxycholate, 1% IGEPAL, pH 7.4, supplemented with protease inhibitor mixture set III, 1 mM phenylmethylsulfonyl fluoride, and 10 mM EDTA. Biotinylated integrin constructs were precipitated from cell lysates using the αVβ3 antibody LM609 (Millipore) and Protein G Agarose. Precipitated samples were solubilized and reduced using 2% SDS –10 mM DTT and analyzed by Western blotting with Extravidin conjugated with horseradish and a TMB/M substrate [53].

### Molecular Modeling of Furin Substrate Complex

A molecular modeling of furin – substrate complex was initiated from the crystal structure of the enzyme bound to the DEC (PDB 1P8J). The peptide substrate SSNSRKRR↓S was modeled using positions of the heavy atoms of the inhibitor and building side chains of amino acids. The short molecular dynamics simulations combined with molecular mechanics minimization were used to optimize substrate atoms. For energy calculations, we used AMBER11 molecular modeling package [22], an ff99SB force field [54] and Generalized-Born method for representing solution environment implicitly [55].

### Functional Pathway Analysis

Functional analysis of proteins with potential furin cleavage sites was performed using Ingenuity Pathway Analysis software using the default parameters (Ingenuity Systems) [32].

## Supporting Information

Table S1 **Raw experimental peptide data.**
(XLS)

Table S2 **Furin protein substrates experimental combined data.**
(XLS)

Table S3 **All predicted furin substrates.**
(XLS)
